# Time-series analysis of daily ambient temperature and emergency department visits in five US cities with a comparison of exposure metrics derived from 1-km meteorology products

**DOI:** 10.1186/s12940-021-00735-w

**Published:** 2021-05-07

**Authors:** Nikita Thomas, Stefanie T. Ebelt, Andrew J. Newman, Noah Scovronick, Rohan R. D’Souza, Shannon E. Moss, Joshua L. Warren, Matthew J. Strickland, Lyndsey A. Darrow, Howard H. Chang

**Affiliations:** 1 Department of Biostatistics and Bioinformatics, Emory University, Atlanta, USA; 2 Gangarosa Department of Environmental Health, Emory University, Atlanta, USA; 3 Research Applications Laboratory, National Center for Atmospheric Research, Boulder, USA; 4 Department of Biostatistics, Yale University, New Haven, USA; 5 School of Community Health Sciences, University of Nevada Reno, Reno, USA

**Keywords:** Temperature, Health effect, Emergency department visits, Exposure assessment, Daymet

## Abstract

**Background:**

Ambient temperature observations from single monitoring stations (usually located at the major international airport serving a city) are routinely used to estimate heat exposures in epidemiologic studies. This method of exposure assessment does not account for potential spatial variability in ambient temperature. In environmental health research, there is increasing interest in utilizing spatially-resolved exposure estimates to minimize exposure measurement error.

**Methods:**

We conducted time-series analyses to investigate short-term associations between daily temperature metrics and emergency department (ED) visits for well-established heat-related morbidities in five US cities that represent different climatic regions: Atlanta, Los Angeles, Phoenix, Salt Lake City, and San Francisco. In addition to airport monitoring stations, we derived several exposure estimates for each city using a national meteorology data product (Daymet) available at 1 km spatial resolution.

**Results:**

Across cities, we found positive associations between same-day temperature (maximum or minimum) and ED visits for heat-sensitive outcomes, including acute renal injury and fluid and electrolyte imbalance. We also found that exposure assessment methods accounting for spatial variability in temperature and at-risk population size often resulted in stronger relative risk estimates compared to the use of observations at airports. This pattern was most apparent when examining daily minimum temperature and in cities where the major airport is located further away from the urban center.

**Conclusion:**

Epidemiologic studies based on single monitoring stations may underestimate the effect of temperature on morbidity when the station is less representative of the exposure of the at-risk population.

**Supplementary Information:**

The online version contains supplementary material available at 10.1186/s12940-021-00735-w.

## Introduction

Studies of the relationship between ambient temperature and adverse health outcomes routinely utilize observations at single airport weather stations to derive exposure estimates. While airport stations have long time series and high-quality data, their measurements may not accurately reflect average exposure experienced by the surrounding study population. Specifically, airport monitors cannot capture intra-urban spatial variability driven by features of the built and natural environment [1, 2], especially when there exist urban-rural gradients due to urban heat island effects [3, 4].

Exposure measurement error that arises from insufficient characterization of exposure spatial variability may lead to attenuated health effect estimates [5, 6]. Previous investigations in air pollution epidemiology have demonstrated that using more spatially-resolved exposure estimates can result in stronger associations compared to using a single air quality monitor in the study region [7–9]. Accurate exposure assessment is an important component of population-based health studies, in part because the health association estimates are frequently used in subsequent risk assessment and health impact analyses [10, 11]. While the use of spatially-resolved exposures has become common in air pollution epidemiology, only a few studies on temperature and mortality have considered heat exposure’s spatial variation in short-term health effect studies [12–14]. Though spatially-resolved estimates have been utilized in studies that focus on spatial exposure contrasts [15–18].

Our study has two objectives. The first is to evaluate the potential benefits of using fine-scale meteorology data products when estimating acute health effects of high temperature, as opposed to a single monitoring station. We are particularly interested in comparing health effect estimates obtained from exposure assessment methods that account for spatial variability and those that assume spatially homogenous exposures. The second objective of this paper is to fill an important knowledge gap on the association between high temperature and morbidity as measured by emergency department (ED) visits. Previous work has predominantly focused on mortality [19–21], and hospitalizations [22–25], and many of the multi-city US morbidity studies have been restricted to the Medicare population ages 65 or above.

To achieve these objectives, we conducted time-series analyses of warm-season daily temperature (maximum and minimum) and all-age ED visits in five US cities from different climatic regions: Atlanta, Los Angeles, Phoenix, Salt Lake City, and San Francisco. To estimate exposures, we used Daymet [26], a publicly available product that provides gridded daily meteorology estimates at 1 km spatial resolution starting in 1980. Daymet data have been found to accurately describe ambient temperature and mean heat index at weather stations across the contiguous United States [27]. Daymet also provides complete spatial coverage over the United States such that spatial variability in at-risk population size can also be incorporated when analyzing aggregated health outcomes [28]. To our knowledge, this is the first study to explore the impact of spatially-resolved exposure variables in estimating short-term temperature-morbidity associations.

## Materials and methods

### Emergency department visit and meteorological data

Multi-year, patient-level ED records were obtained from individual hospitals in the Atlanta metropolitan area (1993–2004) and the Georgia Hospital Association (2005–2012), the Arizona Department of Health Services, Bureau of Public Health Statistics (2008–2016), the California Health and Human Services Agency, Office of Statewide Planning and Development; 2005–2016), and the Utah Department of Health, Office of Health Care Statistics (2005–2016). Records included admission date and International Classification of Diseases (ICD) diagnosis codes. ICD 9th revision (ICD-9) codes were used for ED visits prior to October 1, 2015, followed by the use of ICD 10th revision (ICD-10) codes. Our definition of an ED visit included patients seen in the ED as outpatients and discharged, as well as patients admitted as inpatients from the ED. The analysis was restricted to the warmest 6 months of the year, May to October.

We used both the primary and secondary diagnosis codes to identify ED visits for specific health outcomes that have been found to be associated with high temperature in previous studies [29]. The health outcomes of interest for this study were fluid and electrolyte imbalance (ICD-9: 276, ICD-10: E86-E87), acute renal injury (ICD-9: 584, ICD-10: N17), circulatory disease (ICD-9: 390–459, ICD-10: I00-I99), respiratory disease (ICD-9: 460–519, ICD-10: J00-J99), gastrointestinal infections (ICD-9: 001–009, ICD-10: A00-A09), and heat-related illnesses (ICD-9: 992, ICD-10: T67). For each of these outcomes, ED visits were aggregated over the metropolitan statistical area (MSA) by admission date.

Analytic datasets of daily ED visit counts for each city were obtained by aggregating ED visits by day based on patient residential location in each corresponding MSA. Specifically, an ED visit was included in the study if the patient’s residential ZIP code corresponded to a ZIP code tabulation area (ZCTA) that overlapped with the MSA definition for a city, defined by the U.S. Office of Management and Budget based on contiguous counties. The five MSAs varied in the number of counties (and area size): 20 counties (15,013 km^2^) for Atlanta, 2 counties (11,809 km^2^) for Los Angeles, 2 counties (35,406 km^2^) for Phoenix, 3 counties (23,577 km^2^) for Salt Lake City, and 5 counties (6136 km^2^) for San Francisco. Based on the Köppen-Geiger climate classification [30], the five cities are classified as: Atlanta (humid subtropical), Los Angeles (Mediterranean hot summer), Phoenix (acrid climate), San Francisco (Mediterranean warm/cool summer), Salt Lake City (hot summer continental).

Daily maximum temperature, minimum temperature, and average dewpoint temperature observations were obtained from monitors at the major international airport serving each MSA. We also obtained Daymet (https://daymet.ornl.gov/) gridded surfaces and identified all 1 km × 1 km grid cells that are within the MSA boundary. The Daymet temperature product ingests all surface observations from the Global Historical Climatology Network. The algorithm also performs spatial-temporal interpolation using surrounding observations, as well as information on other weather variables, elevation, and land/water masks. A Daymet grid cell is linked to an MSA if the centroid of the 1 km grid cell falls within any of the MSA’s county boundary. Daymet temperature data were used to develop three different daily exposure temperature metrics: a daily simple spatial average of all 1 km grid cells over each MSA, a daily weighted average based on county population, and a daily weighted average based on ZCTA population. For time-series analysis of aggregated health outcomes, the optimal exposure metric should correspond to the average of exposures of all at-risk individuals. Data on population size were obtained from the US Census at the county-level (1990, 2000, and 2010) and at the ZCTA-level (2000 and 2010). Annual populations were based on linear interpolation of decadal Census data. Daily minimum and maximum temperature from the different exposure assessment methods were also averaged to obtain daily mean temperature.

### Statistical analysis

To estimate the association between temperature and ED visits during warm seasons in each city, we used an over-dispersed Poisson log-linear model. The primary analysis focuses on the associations between same-day (lag 0) temperature and ED visit outcome counts, modeled using natural cubic splines with 4 degrees of freedom (3 equidistant interior knots). While mortality studies have usually utilized longer lags (e.g., 7 days), previous analyses have found that acute effects of heat on ED visits operate on shorter lags [29, 31]. We fitted separate models for each city, each ED visit outcome, each exposure variable (maximum, average or minimum temperature), and each exposure assessment method (airport observations, Daymet simple average, Daymet county population weighted average (PWA), and Daymet ZCTA PWA).

All time-series models included the following variables for confounder adjustments. We controlled for daily mean dew-point temperature using natural cubic splines with 4 degrees of freedom. Long-term and seasonal temporal trends were modeled as a smooth function of day-of-year with 6 degrees of freedom. We added interaction terms between time spline coefficients and indicators for year, resulting in year-specific seasonal trends. For heat-related illnesses, we only included interactions between linear day-of-year and indicators of year due to the smaller number of events. We also included indicators for day-of-the-week, federal holidays, and hospital-specific indicators to account for hospitals’ contributions to the total ED visits in the city.

To better compare the strength of non-linear associations between ED counts and temperature across different exposure assessment methods, we report relative risk based on two different contrasts along the exposure range: 75th percentile versus 25th percentile, and 95th percentile versus 50th percentile. The percentile values were specific to each city and exposure metric. Some studies have defined a common reference temperature by identifying the temperature value that corresponds to the minimum risk on the non-linear function [32]. We opted for a percentile-based method because of the restriction to the warm season and because the range of exposure varied across exposure methods.

It is difficult to statistically assess differences in relative risks estimated from to the use of different exposure assessment methods because these models were fit to the same health outcome and were non-nested. In addition to qualitatively examining the magnitude of associations, we considered two model comparison tools: (1) the Akaike information criterion (AIC) assuming the outcome is Poisson and (2) the estimated overdispersion parameter. Lower values of AIC and overdispersion are preferred, and may indicate better model fit and out-of-sample prediction performance. We note that these are general model comparison tools that do not necessarily reflect the accuracy and precision in inference for the relative risk parameters of interest. We conducted three sensitivity analyses to examine the robustness of the estimated associations and comparison across exposure assessment methods. First, we varied the natural cubic spline’s degrees of freedom for the exposure of interest to 3, 5, or 6. Second, we evaluated an additional exposure assessment method by considering only the Daymet grid cell linked to the airport monitor. Third, we modified the temperature exposure metric to a 3-day moving average (same-day, lag-1, lag-2).

## Results

Descriptive statistics (mean and standard deviation) of the four temperature metrics are given in Table 1. Study locations showed clear geographical differences in temperature. For all exposure metrics, San Francisco and Los Angeles had lower temperatures, while Phoenix had considerably higher temperatures. Supplementary Figure S1 shows the spatial variability in average Daymet maximum and minimum temperature (May to October) at 1 km resolution and locations of airport monitors. We assessed spatial variability by computing the standard deviation of Daymet values within each MSA on each day. Across the study period, the average standard deviation for maximum (minimum) temperature were: 0.33 (0.27) for Atlanta, 1.94 (0.99) for Los Angeles, 2.19 (1.76) for Phoenix, 3.29 (2.13) for Salt Lake City, and 0.79 (0.45) for San Francisco. Greater within-MSA variability was observed for cities with larger area and with more heterogeneous topography.

**Table 1:** Mean (standard deviation) in °C of daily maximum (TMX), mean (AVG) and minimum (TMN) temperature during May to October by study city for four exposure metrics: airport observations, simple average of Daymet 1 km data, and Daymet population-weighted averages (PWA) using county or ZIP code population

City	Exposure	Airport Obs.	Daymet Average	Daymet County PWA	Daymet ZCTA PWA
Atlanta	TMX	28.2 (4.6)	28.6 (4.4)	28.6 (4.3)	28.7 (4.4)
	AVG	22.5 (4.3)	22.8 (4.4)	22.6 (4.3)	23.4 (4.4)
	TMN	16.6 (4.8)	18.6 (4.6)	16.6 (4.8)	16.5 (4.8)
Los Angeles	TMX	23.1 (3.3)	27.5 (4.1)	28.0 (4.3)	24.6 (3.5)
	AVG	21.7 (3.5)	20.5 (2.7)	21.7 (3.8)	19.9 (2.5)
	TMN	15.1 (3.3)	16.6 (2.2)	16.0 (2.6)	15.4 (3.1)
Phoenix	TMX	37.9 (4.9)	37.3 (4.7)	36.0 (4.5)	37.8 (4.7)
	AVG	28.1 (4.6)	30.1 (4.7)	28.7 (4.6)	31.5 (4.7)
	TMN	21.0 (5.0)	25.0 (4.9)	22.1 (5.0)	20.3 (4.9)
Salt Lake City	TMX	27.5 (7.4)	25.5 (6.8)	23.1 (6.6)	27.4 (7.1)
	AVG	15.8 (5.8)	20.3 (6.3)	17.5 (6.0)	20.5 (6.6)
	TMN	10.8 (5.5)	13.5 (6.2)	11.9 (5.6)	8.5 (5.3)
San Francisco	TMX	21.7 (3.7)	23.4 (3.8)	23.9 (3.9)	21.1 (3.3)
	AVG	18.0 (2.8)	16.6 (2.2)	18.5 (3.0)	17.2 (2.5)
	TMN	12.4 (2.2)	12.8 (1.9)	12.3 (2.0)	12.0 (2.1)

Pairwise correlations between the four temperature metrics are given in Supplementary Table S1. The three Daymet metrics were highly correlated (≥ 0.90) with airport observations, except for Los Angeles and San Francisco. For example, correlations between Daymet average and airport observations for minimum temperature were 0.93 for Atlanta, 0.87 for Los Angeles, 0.95 for Phoenix, 0.97 for Salt Lake City, and 0.79 for San Francisco. Airport monitors in Los Angeles and San Francisco are located by the water (Figure S1) and this likely contributed to their weaker correlations with other exposure metrics [33]. We also calculated correlations between on the subset of days when airport monitor observations were above its the 75th percentile. The correlations were consistently lower when restricted to the highest quartiles of temperature, especially for daily maximum temperature in Los Angeles.

**Fig. 1:**
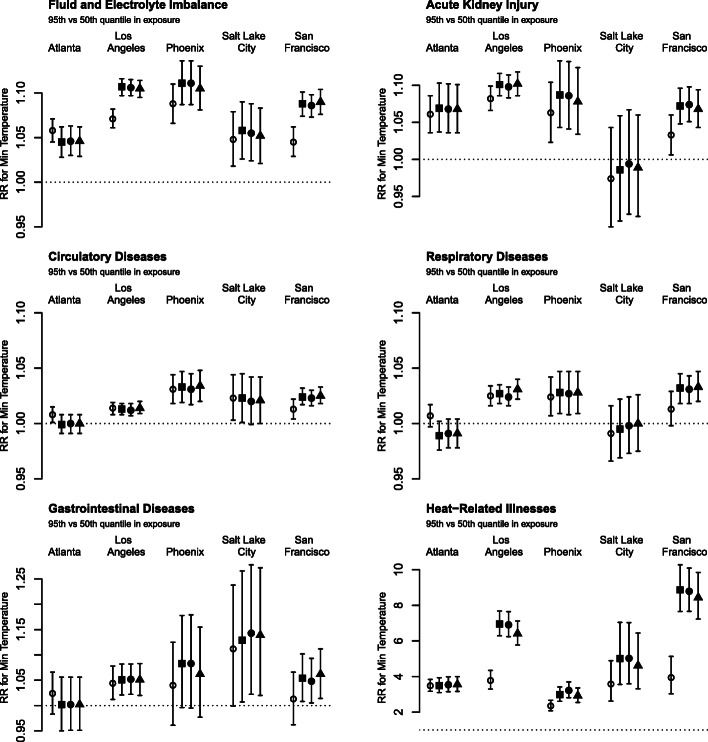
Relative risks (RR) of daily emergency department visits associated with same-day minimum temperature between the 95th and the 50th percentile, comparing four different exposure assessment methods: airport observations (○), Daymet average (◼), Daymet county-level population-weighted average (●), and Daymet ZCTA population-weighted average (▲). The y-axis ranges are different across outcomes

**Fig. 2:**
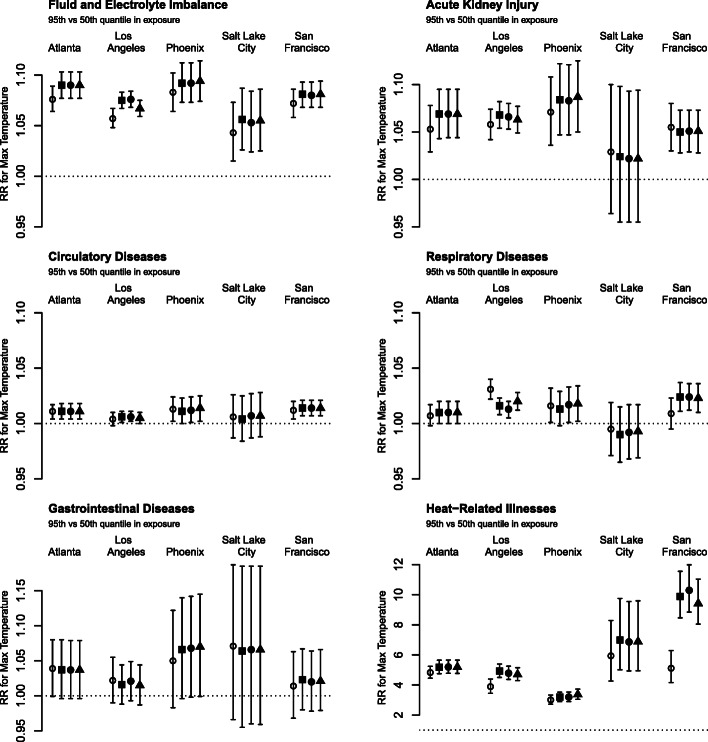
Relative risks (RR) of daily emergency department visits associated with same-day maximum temperature (Max) between the 95th and the 50th percentile, comparing four different exposure assessment methods: airport observation (○), average of Daymet data (◼), county-level population-weighted average (●), and ZIP code-level population-weighted average (▲). The y-axis ranges are different across outcomes

Outcome-specific total and mean daily ED visits are shown in Table 2. Relative risks (RRs) of daily ED visits associated with same-day temperature between the 95th and the 50th percentiles are shown in Fig. 1 for minimum temperature and in Fig. 2 for maximum temperature. Overall, consistent positive associations were identified between ambient temperature and ED visits. We also found evidence that exposure assessment methods accounting for spatial variability in temperature and population are associated with stronger RR estimates compared to the use of airport monitor observations. This pattern is most apparent for Los Angeles and San Francisco, for outcomes that are particularly heat-sensitive (i.e., fluid and electrolyte imbalance, heat-related illnesses, and acute kidney injury), and for minimum temperature. Similar patterns were found for RRs between the 75th and 25th percentile of the exposure (Supplementary Figures S2 and S3). RRs for daily mean temperature (Supplemental Figures S4 and S5) are similar to RRs or daily maximum temperature. We continue to see stronger associations with the use of Daymet data projects in Los Angeles and San Francisco, and for fluid and electrolyte imbalance and heat-related illnesses.

**Table 2:** Total counts of emergency department visits during May to October in five US cities. Daily average counts are provided in parentheses

	Atlanta 1993–2012	Los Angeles 2005–2016	Phoenix 2008–2016	San Francisco 2005–2016	Salt Lake City 2005–2016
Fluid and electrolyte imbalance	593,202 (162)	1,447,202 (655)	321,448 (194)	548,596 (248)	108,112 (49)
Acute renal injury	131,444 (36)	392,949 (179)	70,735 (8)	139,685 (63)	17,035 (8)
Circulatory diseases	2,289,447 (622)	4,637,289 (2100)	875,401 (529)	1,909,559 (865)	229,422 (104)
Respiratory diseases	1,971,681 (536)	3,130,345 (1418)	715,187 (432)	1,315,687 (596)	193,141 (87)
Gastrointestinal infections	54,712 (15)	95,024 (43)	17,151 (10)	37,574 (17)	8406 (4)
Heat-related illness	12,332 (3)	9484 (4)	5986 (4)	3081 (1)	685 (0)

**Fig. 3:**
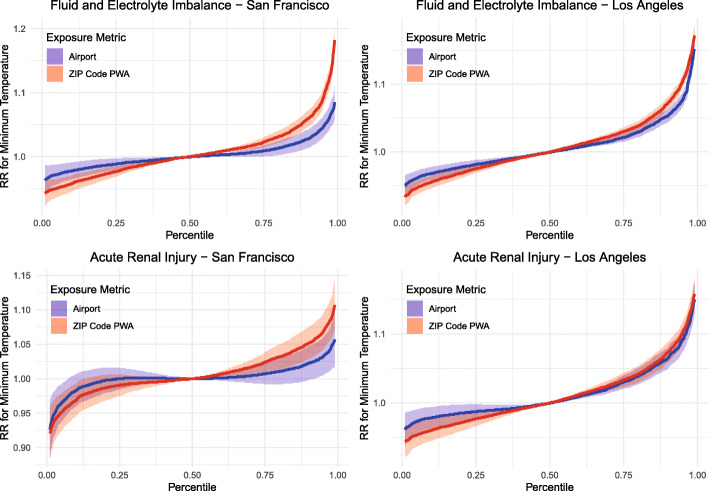
Exposure-response functions and 95% pointwise confidence intervals for warm-season daily minimum temperature and same-day emergency department visits for fluid and electrolyte imbalance and acute kidney injury, comparing two different exposure assessment methods: airport observations and Daymet ZCTA population-weighted average (PWA). Reference level for the relative risk (RR) is the median observed temperature value for each exposure metric

To better visualize differences in the entire exposure-response function due to exposure assessment methods, Fig. 3 shows the relative risk for two outcomes (fluid and electrolyte imbalance, and acute kidney injury) in Los Angeles and San Francisco, comparing the airport observations and Daymet ZCTA PWA exposures. The exposure-response function is centered at the 50th percentile of each exposure metric. We see evidence that accounting spatial variability resulted in steeper changes in relative risks across the entire exposure distribution in the cities with the largest differences in temperature distribution when comparing their Daymet exposures and airport observations. Differences in AIC and ratios of overdispersion for each city, outcome, and temperature metric are given in Supplementary Table S3. In general, we see evidence that the use of Daymet-derived exposures resulted in smaller AIC and overdispersion in Los Angeles and San Francisco. This suggests that the differences observed in RRs and exposure-response relationships lead to better model fit.

In sensitivity analyses, when considering using Daymet values at the airport monitors, we found that estimated RRs were either similar to those using airport observations (e.g., Los Angeles and San Francisco) or similar to those using population-weighted Daymet data (Supplementary Figures S4 and S5). The estimated RRs across exposure metrics were also robust against changing the degrees of freedom for the exposure-response function from 4 to 3, 5, or 6 (results for Daymet ZCTA PWA exposures given in Supplementary Figures S6 and S7). RRs of daily ED visits associated with 3-day moving-average temperature between the 95th and the 50th are given in Supplementary Figures S8 and S9. Generally, we found that the use of moving average temperature data reduced differences between using airport observations and Daymet ZCTA PWA exposures. One explanation may be that additional temporal smoothing increased the correlation between monitor measurements and Daymet-derived exposures. However, differences in relative risk estimates across exposure metrics were still more pronounced for minimum temperature and in San Francisco and Los Angeles. Numerical values of all estimated relative risks and 95% confidence intervals for all primary analyses are provided in the Supplementary Materials.

## Discussion

We conducted time-series analyses to examine associations between same-day temperature and ED visits in five US cities: Atlanta, Los Angeles, Phoenix, Salt Lake City and San Francisco. In addition to airport monitoring stations, we utilized the Daymet maximum and minimum temperature products at 1 km spatial resolution. Overall, we found consistent positive associations between ambient temperature and ED visits for various outcomes across cities, though associations were often stronger when using Daymet-based exposures as compared to those from the airport stations. Furthermore, incorporating spatial distributions of the at-risk population may provide a more accurate measure of the temperature experienced by the populations of each of the cities.

A few recent temperature and mortality studies have also considered spatially-varying temperature estimates developed by various modeling approaches. For example, a study in Brisbane, Australia showed that spatially-resolved exposure estimates obtained from geostatistical models gave a slightly better model fit and stronger exposure-response functions compared to observations at a central monitor or averages of multiple monitors [12]. Similar findings are reported by Lee et al. [13], in a study in Southeastern US with 1 km exposure estimated using a statistical model with satellite parameters. Finally, in a study of 113 US counties, Weinberger et al. [14], found that associations were largely similar with the use of weather station observations or population-weighted temperature estimates based on a 4 km temperature product. To our knowledge, our study is the first to compare impacts of different temperature exposure metrics using spatially-resolved temperature data products in analyzing ED visits instead of mortality.

Another unique aspect of our study is the evaluation of daily minimum temperature. Prolonged exposure to heat can overwork the body’s natural compensatory mechanisms. Studies have shown that hot nights measured by daily minimum temperature preceded or followed by hot days are associated with adverse health outcomes and excess mortality [34–36]. This has been attributed to the body not having time to recover from heat exposure. In order to maintain thermal homeostasis during heat events, cardiac output increases and the body redirects blood flow from vital organs to the skin, thus cooling the body [37]. Sweating also increases to cool the body, but excessive sweating can lead to reduced blood volume and dehydration. These mechanisms are often attenuated in the elderly or populations with comorbidities and could be a contributing factor to the increases seen in mortality risk when using a minimum temperature threshold [36, 38]. Murage et al. [36], found mortality risks were increased on hot nights followed by a hot day more than on cool nights followed by hot days. Overall, the above studies examined minimum temperature and mortality risks but could help explain the strong associations seen with minimum temperatures and morbidity for this analysis. We also note that maximum daily temperature typically occurs after half of the day has elapsed. Hence the weaker observed associations may reflect the temporal misalignment between outcome and short-term exposure. In particular, Davis et al. [39], examined associations between hourly temperature and mortality, and found that yesterday’s afternoon temperature had strong associations in Boston, Philadelphia and Seattle. Hence, future work may consider exploring the use of Daymet products in estimating exposure lag structures for different outcomes and temperature characteristics.

We found that differences in estimated RR’s between airport observations and Daymet-based exposures to be larger for minimum temperature than maximum temperature. One possible explanation is that minimum temperature corresponds to night-time exposure when individuals are most likely to be at home. Hence, using population-weighted average may better reflect average exposures. In contrast, population-weighted maximum temperature exposure may still be subject to considerable error due to variation in between-individual time-activity patterns. Another possible explanation is that urbanization has stronger impacts on night time temperature compared to daytime hours, contributing to larger spatial variation in minimum temperature that may not be captured by airport monitors. We also note that distributions of Daymet-based minimum temperatures exposures were more different than airport observations (Table S2). For example, in Los Angeles, the differences between the 95th and 50th percentile were 4.25 °C for Daymet ZCTA population-weighted average versus 3.33 °C for airport observations. Hence, the larger absolute change in exposure may also contribute to higher risk estimates.

Although Daymet is a well-established meteorology product, one limitation is its incomplete representation of urban heat islands (UHIs), which comes from several factors. First, Daymet uses stations compiled within the Global Historical Climatology Network-Daily (GHCN-D) dataset, from the US National Centers for Environmental Information. For the US, the GHCN-D dataset compiles US automated surface observing stations (ASOS) which include airports, cooperative network stations (e.g. US National Weather Service cooperative network stations), and some regional mesonets (e.g. Remote Automatic Weather Station [RAWS],), providing roughly 10–15,000 temperature observations a day across the contiguous US through the study period [40], which may have insufficient density in to fully observe UHIs. Additionally, Daymet uses a truncated Gaussian filter to perform the spatial interpolation of the station observations to the grid (on average 20 stations per grid point), effectively smoothing small scale observed variations in regions of high observation density. Finally, Daymet uses elevation as a predictor within their model (as elevation strongly influences temperature), but does not use any other geophysical attributes such as urban fraction [26]. These three factors limit the representation of UHIs in Daymet to resemble smooth bullseyes with possibly reduced magnitudes. We note that the generalized concepts of sparse stations, interpolation functions, and limited geophysical predictors are common to essentially all currently available in situ observation based gridded meteorology products.

Possible extensions to alleviate these concerns include use of supplemental station networks, use of other geophysical attributes as predictors in statistical models, or incorporation of urban models within the general interpolation framework to explicitly represent UHIs. For example, there are many thousands more stations available in the past 10–15 years through local mesonets that may improve representation of UHIs. There are also high-resolution land use/land cover maps that allow for inclusion of other geophysical predictors in a statistical model, or use of physically based urban models [41], that explicitly model the full energy balance of urban grid cells to better represent the spatial variability of UHIs [42]. These extensions are the subject of current research by this team [43].

Our study also has several limitations common to those that utilize administrative health databases. First, our exposure estimates do not account for individual activity patterns and indoor temperature. However, by using only temporal contrast in exposure, long-term trends that impact personal exposures should not confound estimated relative risks. Moreover, quantifying risks associated with ambient temperature is more relevant for designing local heat warning systems. Second, we did not control for ambient air pollution, such as ozone and fine particulate matter, which have been associated with various cardiorespiratory outcomes. Higher temperature often increases pollutant concentrations due to increased emission (e.g., from electricity generation) and favorable conditions for pollutant formation and transport. Hence, controlling for ambient pollution may reduce the total association of temperature on ED visits [44]. However, some multi-city mortality studies have also demonstrated that adjusting for ambient air pollution often lead to similar health effect estimates for temperature [20, 45, 46]. Finally, our empirical analyses do not allow for a rigorous quantification of attenuation in estimated relative risks due to spatial variation in heat exposures. A simulation study will help characterize how different degrees of spatial variation and the location of the single observation monitor can impact health effect estimation. The 1 km spatial resolution of Daymet can also be used to investigate whether this is an optimal spatial aggregation most practical for short-term exposure and health studies.

## Conclusion

In summary, this study found positive health associations between high temperature and emergency department visits in five US cities located in different climate regions. The comparison of different exposure metrics suggests that epidemiological studies based on single monitoring stations may underestimate the effect of temperature on morbidity when monitoring station is less representative of the exposure of the at-risk population. Our results further demonstrate the potential advantages of using spatially-resolved, population-weighted exposure estimates in estimating health effects of short-term heat exposure, and can benefit from future studies that examine additional locations, health outcomes, and exposure lag structures.

## Supplementary Information

**Additional file 1: Table S1.** Descriptive statistics of exposures by city, temperature metric and exposure assessment methods. **Table S2.** Quantile values of exposures by city and exposure metrics. **Table S3.** Difference in AIC and ratio of overdispersion by city, outcome and temperature exposure. **Figure S1.** Maps of average May-October daily Daymet 1km temperature estimates by city. **Figure S2.** Associations with minimum temperature between the 75th and 25th percentile of exposures. **Figure S3.** Associations with maximum temperature between the 75th and 25th percentile of exposures. **Figure S4.** Associations with average temperature between the 95th and 50^h^ percentile of exposures. **Figure S5.** Associations with average temperature between the 75th and 25^h^ percentile of exposures. **Figure S6.** Associations with minimum temperature with Daymet grid cell link to the monitor. **Figure S7.** Associations with maximum temperature with Daymet grid cell link to the monitor. **Figure S8**. Sensitivity analyses for different natural cubic spline degrees of freedom for minimum temperature. **Figure S9.** Sensitivity analyses for different natural cubic spline degrees of freedom for maximum temperature. **Figure S10.** Associations for 3-day moving average of minimum temperature between the 95th and 50th percentile of exposures. Figure **S11.** Associations for 3-day moving average of maximum temperature between the 95th and 50th percentile of exposures. **Primary.csv** Model results for primary analysis for same-day exposure (by city, outcome, temperature metric, exposure assessment methods, AIC, overdispersion, and exposure contrast referenced at the 50th exposure percentile). **Primary.csv** Model results for primary analysis for same-day exposure (by city, outcome, temperature metric, exposure assessment methods, AIC, overdispersion, and exposure contrast referenced at the 50th exposure percentile). **Primary_Ref25.csv** Model results for primary analysis for same-day exposure (by city, outcome, temperature metric, exposure assessment methods, AIC, overdispersion, and exposure contrast referenced at the 25th exposure percentile). **PrimaryMV.csv** Model results for primary analysis for 3-day moving average exposure (by city, outcome, temperature metric, exposure assessment methods, AIC, overdispersion, and exposure contrast referenced at the 50th exposure percentile).

## Data Availability

Temperature exposure data are publicly available from online databases. Health data can be requested from state-specific data custodians by establishing appropriate data use agreements.
