# Supplementary material for: Time-series analysis of daily ambient temperature and emergency department visits in five US cities with a comparison of exposure metrics derived from 1-km meteorology products
Source: Environ Health. 2021 May 7;20:55. doi: 10.1186/s12940-021-00735-w (PMC8106140; doi:10.1186/s12940-021-00735-w)
Supplement: Supplementary file 1 — Additional file 1: Table S1. Descriptive statistics of exposures by city, temperature metric and exposure assessment methods. Table S2. Quantile values of exposures by city and exposure metrics. Table S3. Difference in AIC and ratio of overdispersion by city, outcome and temperature exposure. Figure S1. Maps of average May-October daily Daymet 1km temperature estimates by city. Figure S2. Associations with minimum temperature between the 75th and 25th percentile of exposures. Figure S3. Associations with maximum temperature between the 75th and 25th percentile of exposures. Figure S4. Associations with average temperature between the 95th and 50h percentile of exposures. Figure S5. Associations with average temperature between the 75th and 25h percentile of exposures. Figure S6. Associations with minimum temperature with Daymet grid cell link to the monitor. Figure S7. Associations with maximum temperature with Daymet grid cell link to the monitor. Figure S8. Sensitivity analyses for different natural cubic spline degrees of freedom for minimum temperature. Figure S9. Sensitivity analyses for different natural cubic spline degrees of freedom for maximum temperature. Figure S10. Associations for 3-day moving average of minimum temperature between the 95th and 50th percentile of exposures. Figure S11. Associations for 3-day moving average of maximum temperature between the 95th and 50th percentile of exposures. Primary.csv Model results for primary analysis for same-day exposure (by city, outcome, temperature metric, exposure assessment methods, AIC, overdispersion, and exposure contrast referenced at the 50th exposure percentile). Primary.csv Model results for primary analysis for same-day exposure (by city, outcome, temperature metric, exposure assessment methods, AIC, overdispersion, and exposure contrast referenced at the 50th exposure percentile). Primary_Ref25.csv Model results for primary analysis for same-day exposure (by city, outcome, temperature metric, exp [file 12940_2021_735_MOESM1_ESM.docx]

**Supplementary Tables and Figures for**

Time-series analysis of daily ambient temperature and emergency department visits in five US cities: a comparison of exposure metrics derived from 1-km meteorology products

Nikita Thomas, Andrew J Newman, Noah Scovronick, Rohan R D’Souza, Shannon Moss, Joshua L Warren, Lyndsey A Darrow, Matthew J Strickland, Stefanie Ebelt, Howard H Chang*

**Supplementary Table S1.** Daily maximum (TMX), minimum (TMN) and average (AVG) temperature correlations between Daymet exposure metrics and observations at airports during May to October by study city. Correlations calculated only on days where airport monitor observations were above 75^th^ percentile are given in parentheses. The three Daymet metrics are: average of Daymet 1km product and Daymet population-weighted average (PWA) using county or ZIP code (ZCTA) population.

| **Study Location** | **Exposure** | **Daymet Average** | **Daymet County PWA** | **Daymet ZCTA PWA** |
| --- | --- | --- | --- | --- |
| Atlanta | TMX | 0.93 (0.72) | 0.94 (0.74) | 0.94 (0.73) |
| Atlanta | AVG | 0.96 (0.75) | 0.96 (0.76) | 0.96 (0.76) |
| Atlanta | TMN | 0.95 (0.95) | 0.95 (0.95) | 0.95 (0.95) |
|  |  |  |  |  |
| Los Angeles | TMX | 0.71 (0.34) | 0.64 (0.22) | 0.83 (0.60) |
| Los Angeles | AVG | 0.80 (0.42) | 0.75 (0.28) | 0.89 (0.67) |
| Los Angeles | TMN | 0.87 (0.87) | 0.84 (0.84) | 0.93 (0.93) |
|  |  |  |  |  |
| Phoenix | TMX | 0.97 (0.84) | 0.98 (0.88) | 0.98 (0.91) |
| Phoenix | AVG | 0.98 (0.87) | 0.98 (0.90) | 0.98 (0.92) |
| Phoenix | TMN | 0.96 (0.96) | 0.96 (0.96) | 0.97 (0.97) |
|  |  |  |  |  |
| Salt Lake City | TMX | 0.98 (0.85) | 0.99 (0.88) | 0.98 (0.88) |
| Salt Lake City | AVG | 0.99 (0.88) | 0.99 (0.90) | 0.99 (0.89) |
| Salt Lake City | TMN | 0.98 (0.98) | 0.98 (0.98) | 0.98 (0.98) |
|  |  |  |  |  |
| San Francisco | TMX | 0.81 (0.75) | 0.79 (0.71) | 0.86 (0.81) |
| San Francisco | AVG | 0.81 (0.80) | 0.78 (0.77) | 0.86 (0.85) |
| San Francisco | TMN | 0.78 (0.78) | 0.72 (0.72) | 0.82 (0.82) |

**Supplementary Table S2.** Quantile values of daily maximum (TMX), minimum (TMN) and average (AVG) temperature (°C**)** for five exposure metrics during May to October by study city. The five metrics are airport monitor observation, average of Daymet 1km product, Daymet population-weighted average (PWA) using county or ZIP code (ZCTA) populatio, and Daymet grid cell linked to the airport.

**Supplementary Table S3.** Difference in AIC and ratio of overdispersion for each city, outcome, and temperature exposure [daily maximum (TMX), minimum (TMN) and average (AVG) temperature (°C)]. Exposure assessment methods with the minimum AIC or overdispersion are used as the reference and are highlighted. The four exposure assessment methods are airport monitor, Daymet average, Daymet county-level population-weighted average (PWA), and Daymet ZCTA population-weighted average (PWA).

**Supplementary Table S3 (Cont.)**

**Supplementary Figure S1.** Average May-October daily Daymet 1km maximum and minimum temperatures. Airport locations are given by the black triangle. The five MSAs varied in the number of counties (and area size): 20 counties (15,013 km2) for Atlanta, 2 counties (11,809 km2) for Los Angeles, 2 counties (35,406 km2) for Phoenix, 3 counties (23,577 km2) for Salt Lake City, and 5 counties (6,136 km2) for San Francisco.


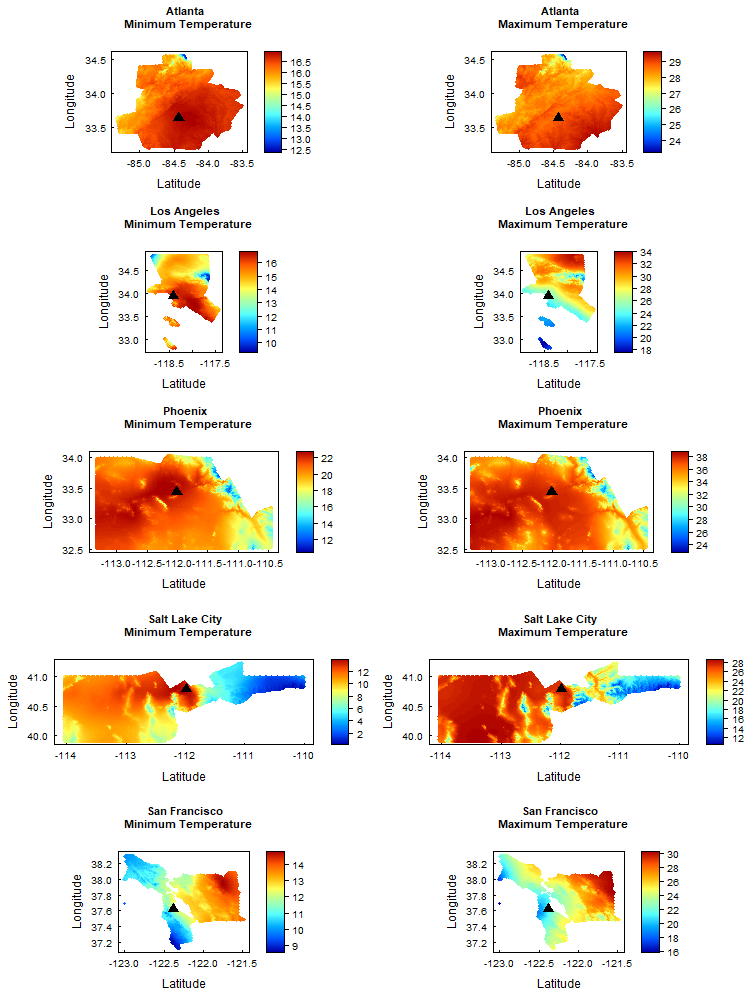


**Supplementary Figure S2.** Relative risks of daily emergency department visits associated with same-day **minimum temperature** (Min) between the **75th and the 25th percentile**, comparing four different exposure assessment methods: airport observation (○), average of Daymet data (◼), county-level population-weighted average (●), and ZCTA population-weighted average (▲). The y-axis ranges are different across outcomes.


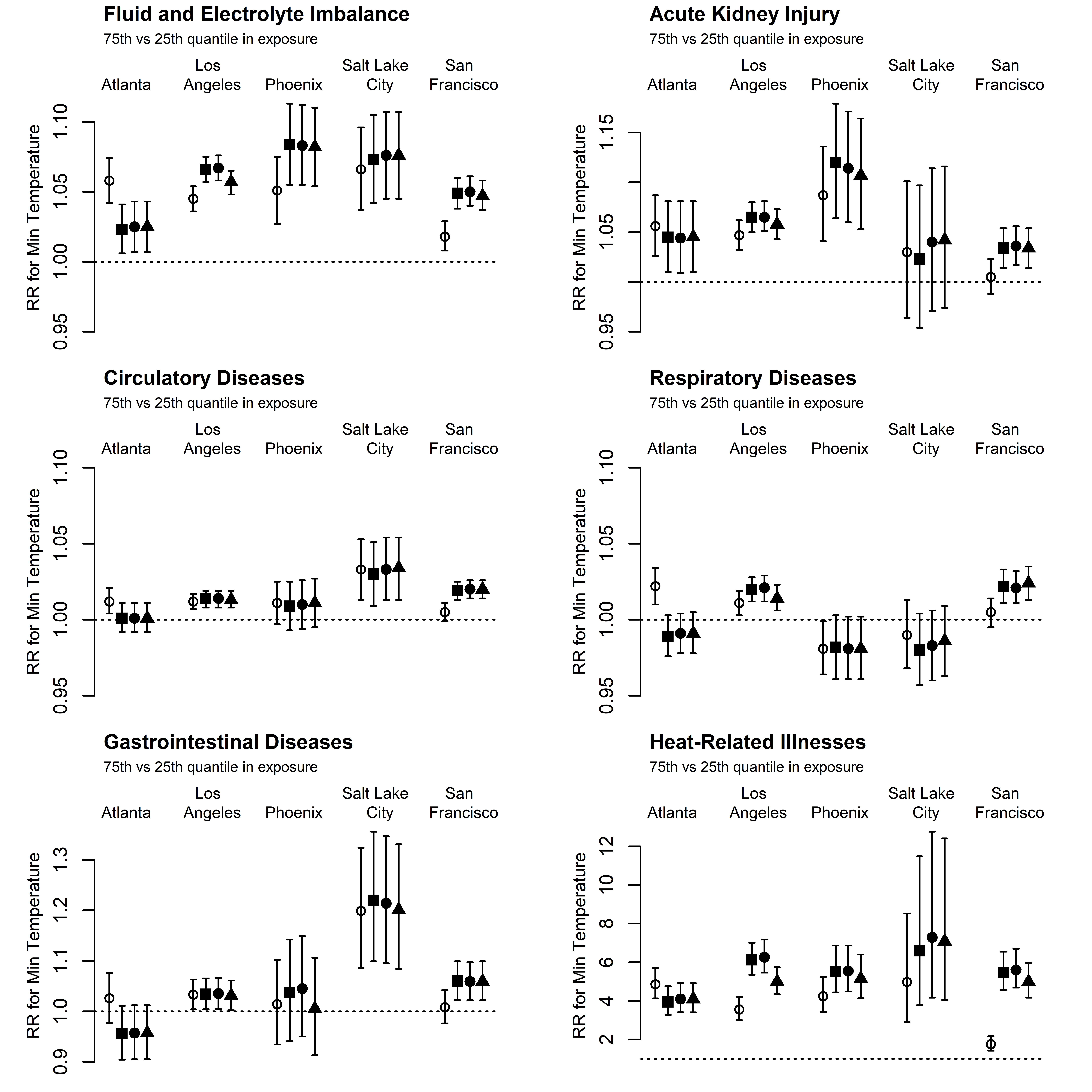


**Supplementary Figure S3.** Relative risks of daily emergency department visits associated with same-day **maximum temperature** between the **75th and the 25th percentile**, comparing four different exposure assessment methods: airport observation (○), average of Daymet data (◼), county-level population-weighted average (●), and ZCTA population-weighted average (▲). The y-axis ranges are different across outcomes.


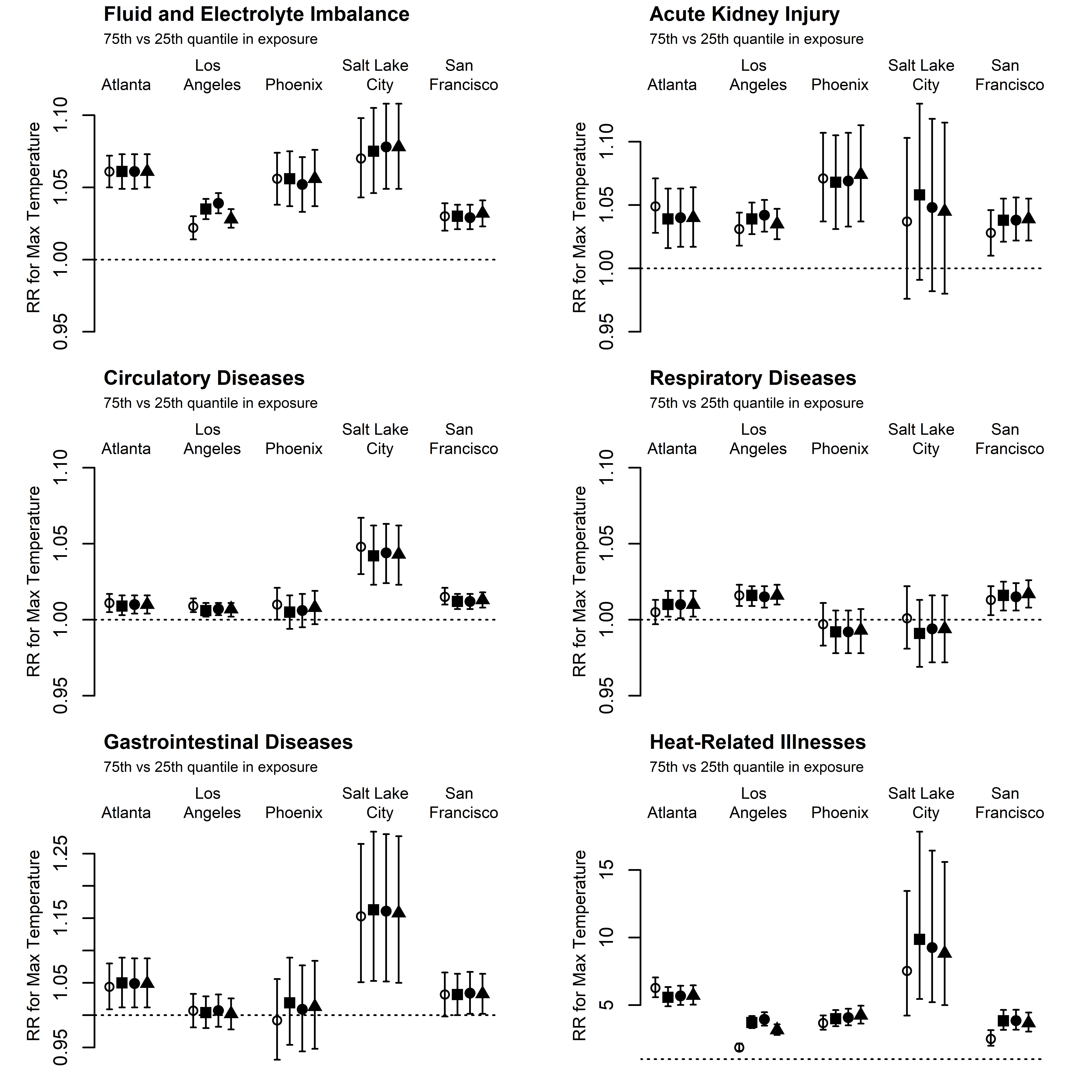


**Supplementary Figure S4.** Relative risks of daily emergency department visits associated with same-day **average temperature** (Avg) between the **95th and the 50th percentile**, comparing four different exposure assessment methods: airport observation (○), average of Daymet data (◼), county-level population-weighted average (●), and ZCTA population-weighted average (▲). The y-axis ranges are different across outcomes.

**
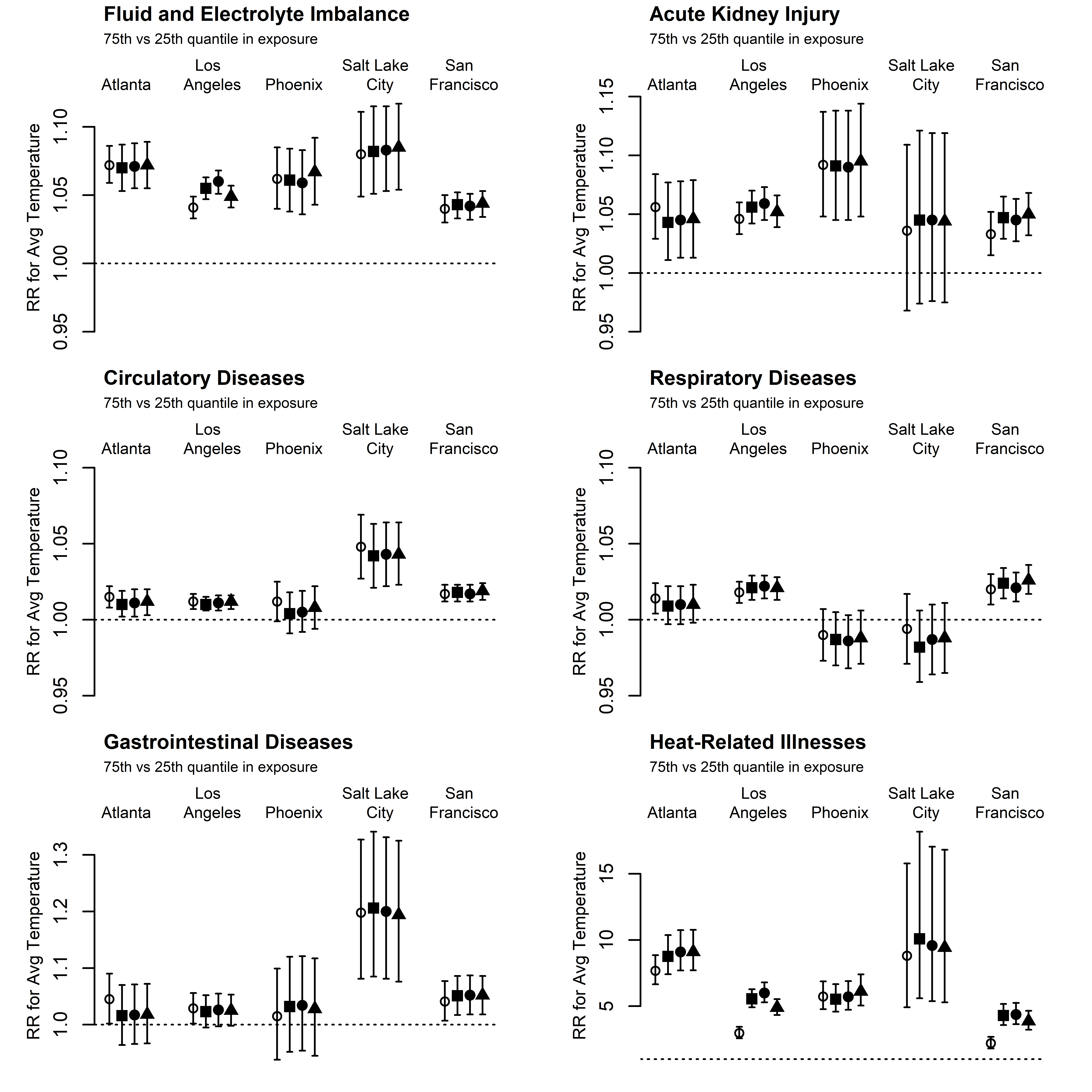
**

**Supplementary Figure S5.** Relative risks of daily emergency department visits associated with same-day **average temperature** (Avg) between the **75th and the 25th percentile**, comparing four different exposure assessment methods: airport observation (○), average of Daymet data (◼), county-level population-weighted average (●), and ZCTA population-weighted average (▲). The y-axis ranges are different across outcomes.

**
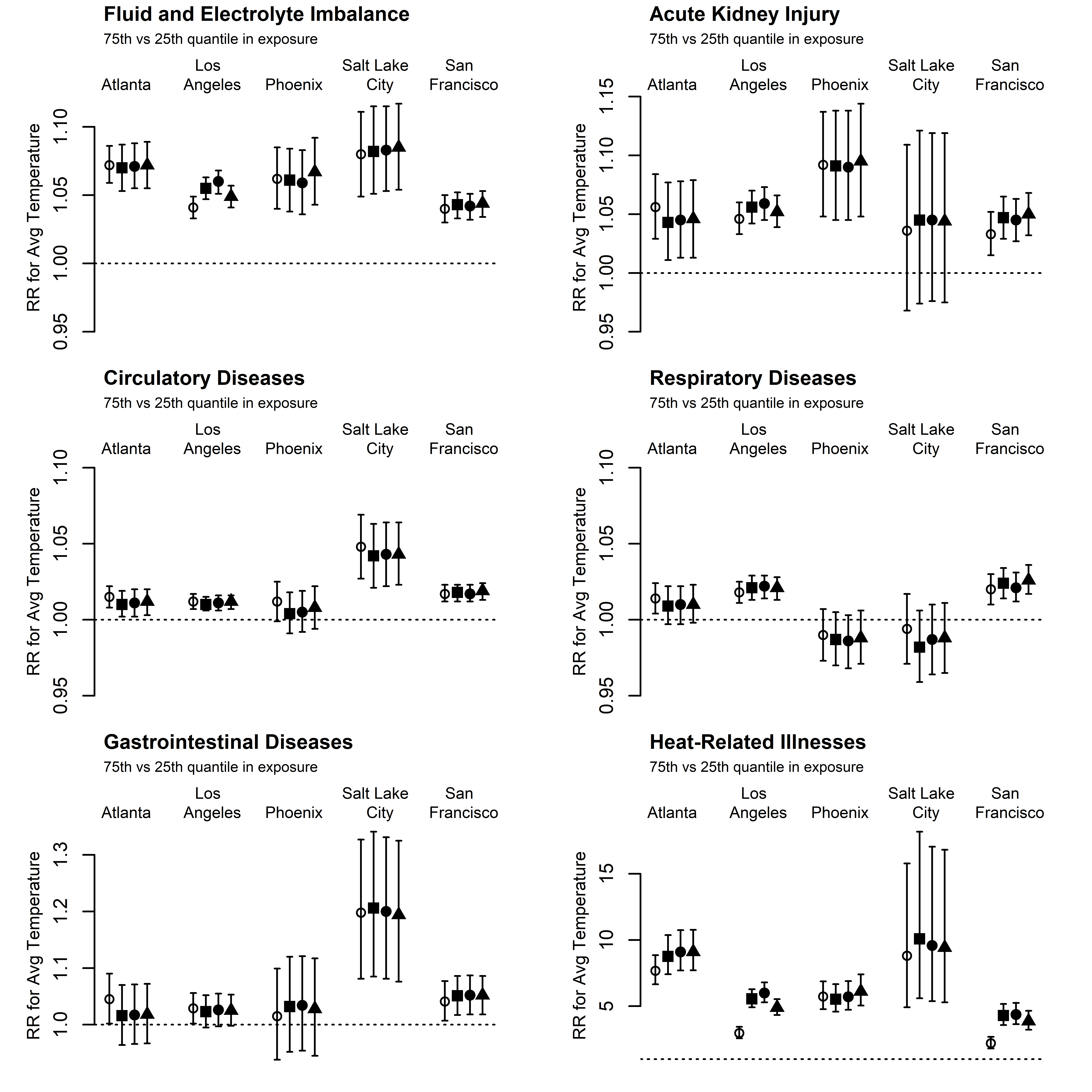
**

**Supplementary Figure S6.** Relative risks of daily emergency department visits associated with same-day **minimum temperature** between the **95th and the 50th percentile**, comparing three different exposure assessment methods: airport observation (○), ZCTA population-weighted average (▲), and Daymet grid cell linked to the monitor (×). The y-axis ranges are different across outcomes.

**
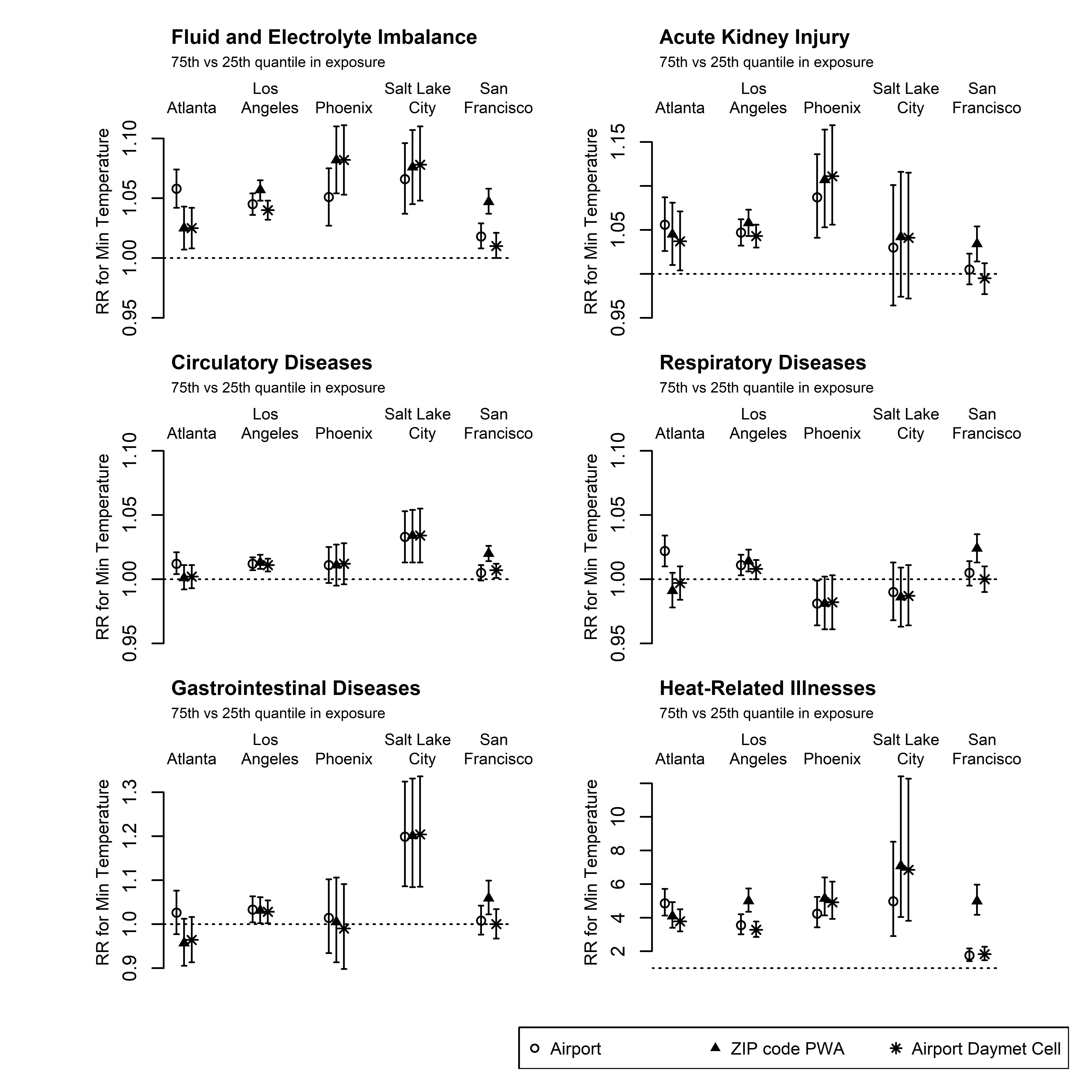
**

**Supplementary Figure S7.** Relative risks of daily emergency department visits associated with same-day **maximum temperature** between the **95th and the 50th percentile**, comparing three different exposure assessment methods: airport observation (○), ZCTA population-weighted average (▲), and Daymet grid cell linked to the monitor (×). The y-axis ranges are different across outcomes.

**
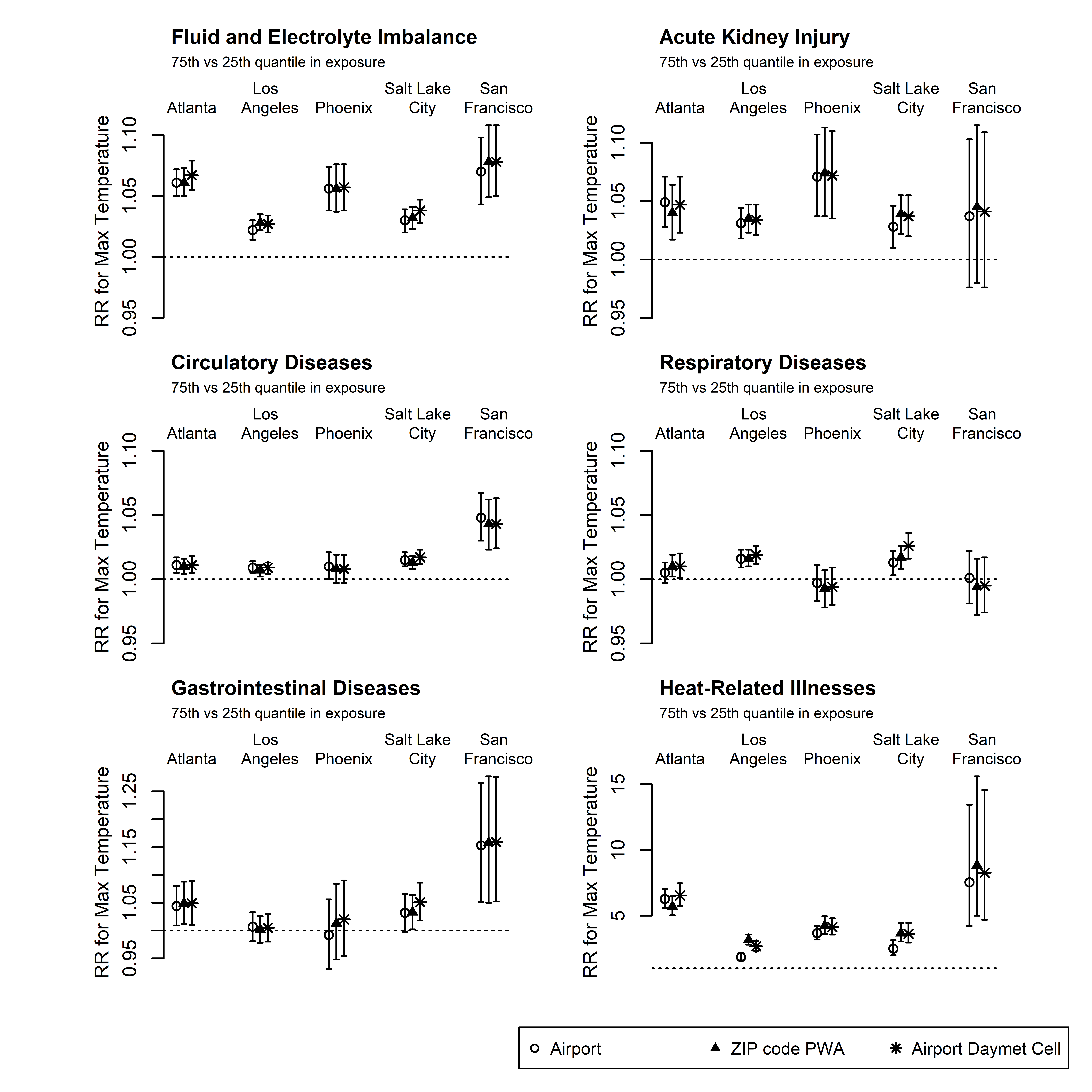
**

**Supplementary Figure S8.** Relative risks (RR) of daily emergency department visits associated with same-day **minimum temperature** between the **95th and the 50th percentile** using the Daymet ZCTA population-weighted average across different natural cubic spline degrees of freedom.

**
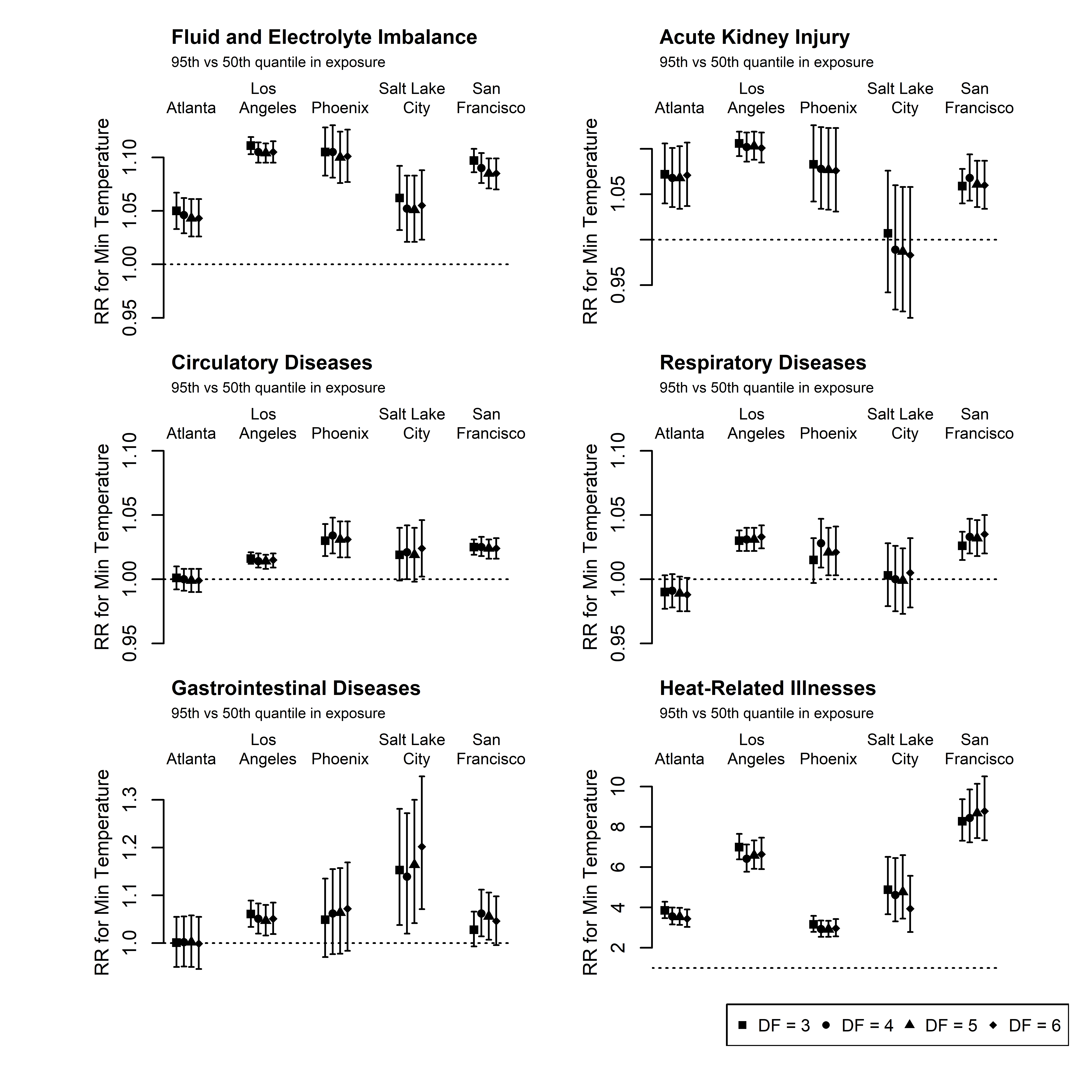
**

**Supplementary Figure S9.** Relative risks (RR) of daily emergency department visits associated with same-day **maximum temperature** between the **95th and the 50th percentile** using the Daymet ZCTA population-weighted average across different natural cubic spline degrees of freedom.

**
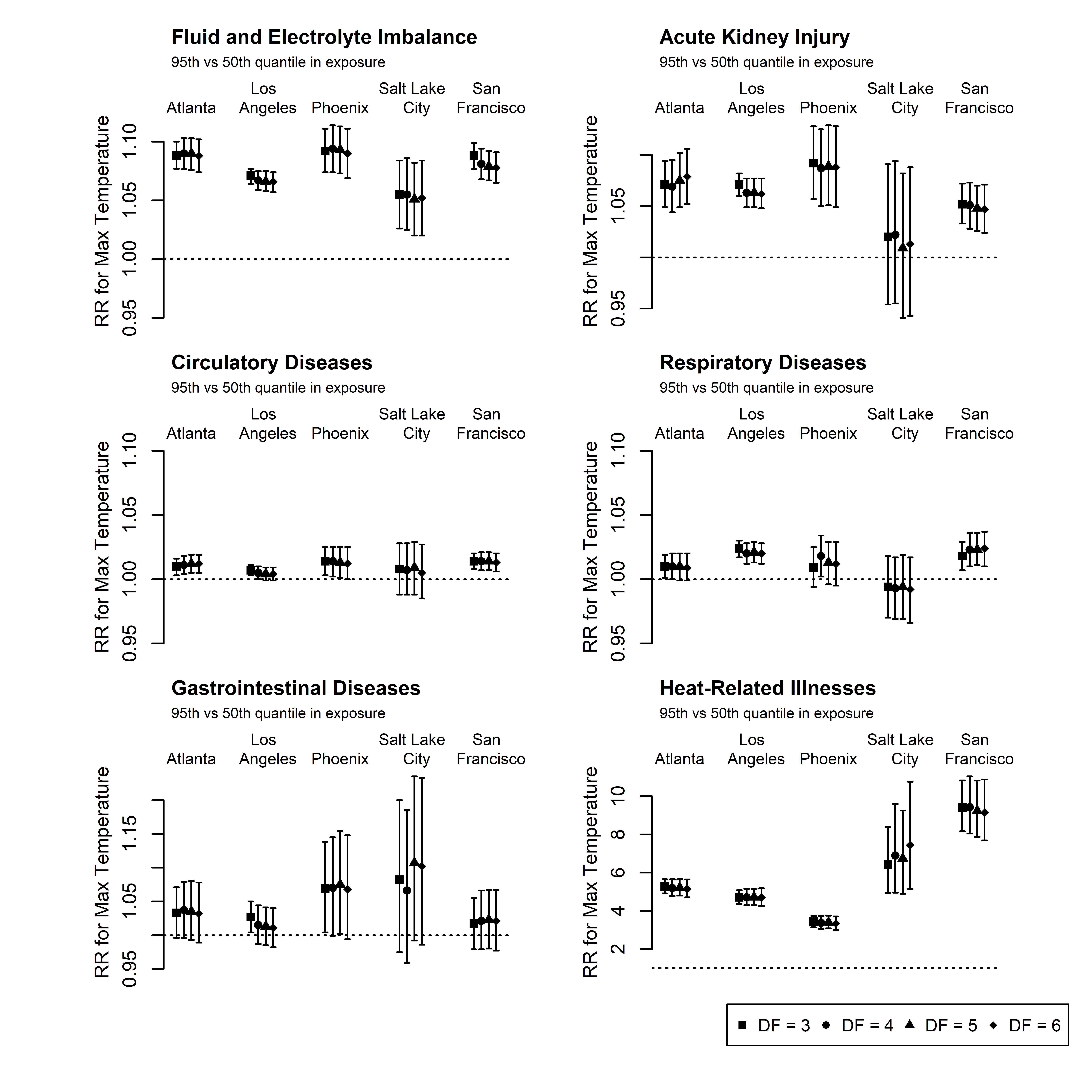
**

**Supplementary Figure S10.** Relative risks (RR) of daily emergency department visits associated with 3-day moving average (lags 0, 1, and 2) of **minimum temperature** between the **95th and the 50th percentile**, comparing four different exposure assessment methods: airport observation (○), average of Daymet data (◼), county-level population-weighted average (●), and ZCTA population-weighted average (▲). The y-axis ranges are different across outcomes.

**
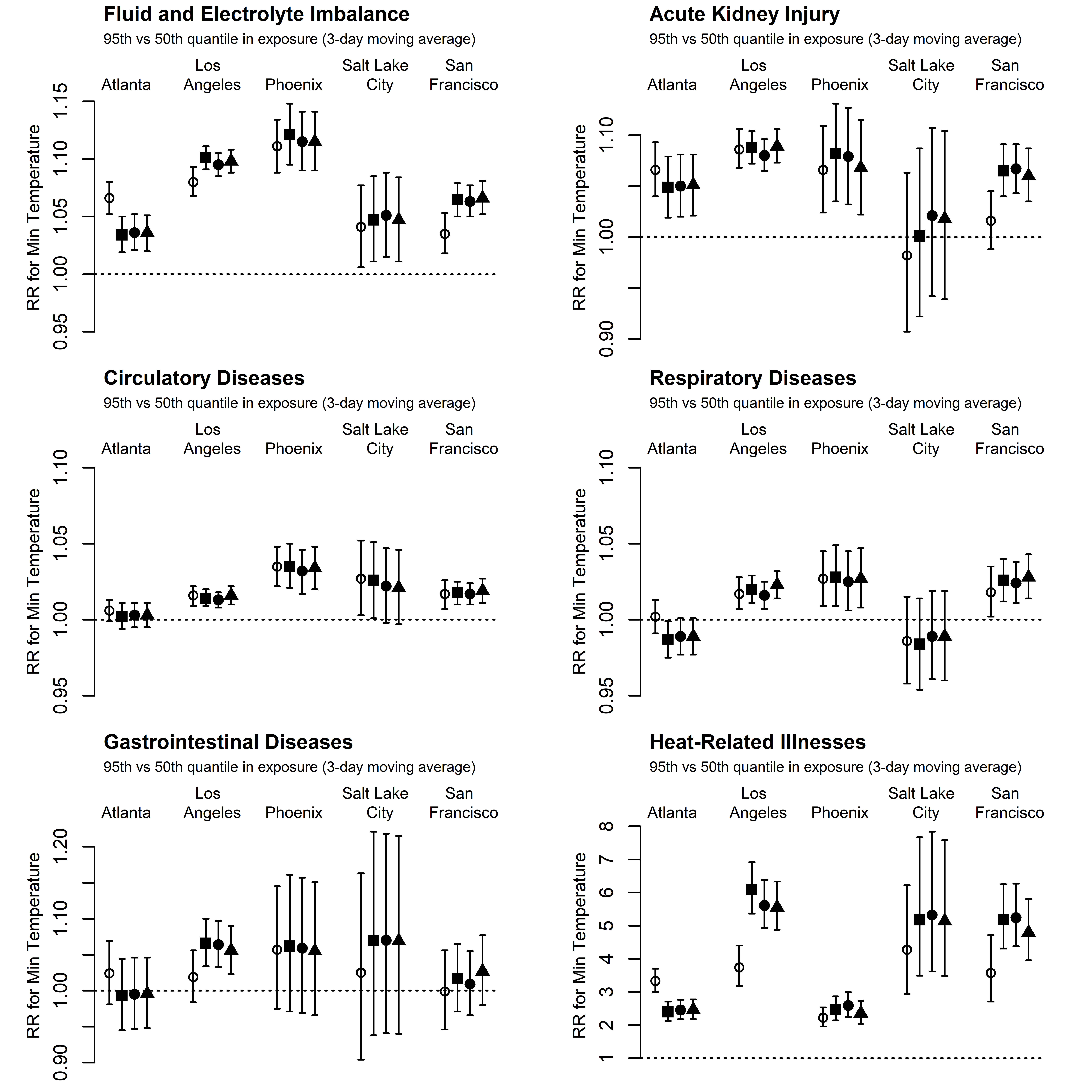
**

**Supplementary Figure S11.** Relative risks (RR) of daily emergency department visits associated with 3-day moving average (lags 0, 1, and 2) of **maximum temperature** between the **95th and the 50th percentile**, comparing four different exposure assessment methods: airport observation (○), average of Daymet data (◼), county-level population-weighted average (●), and ZCTA population-weighted average (▲). The y-axis ranges are different across outcomes.

**
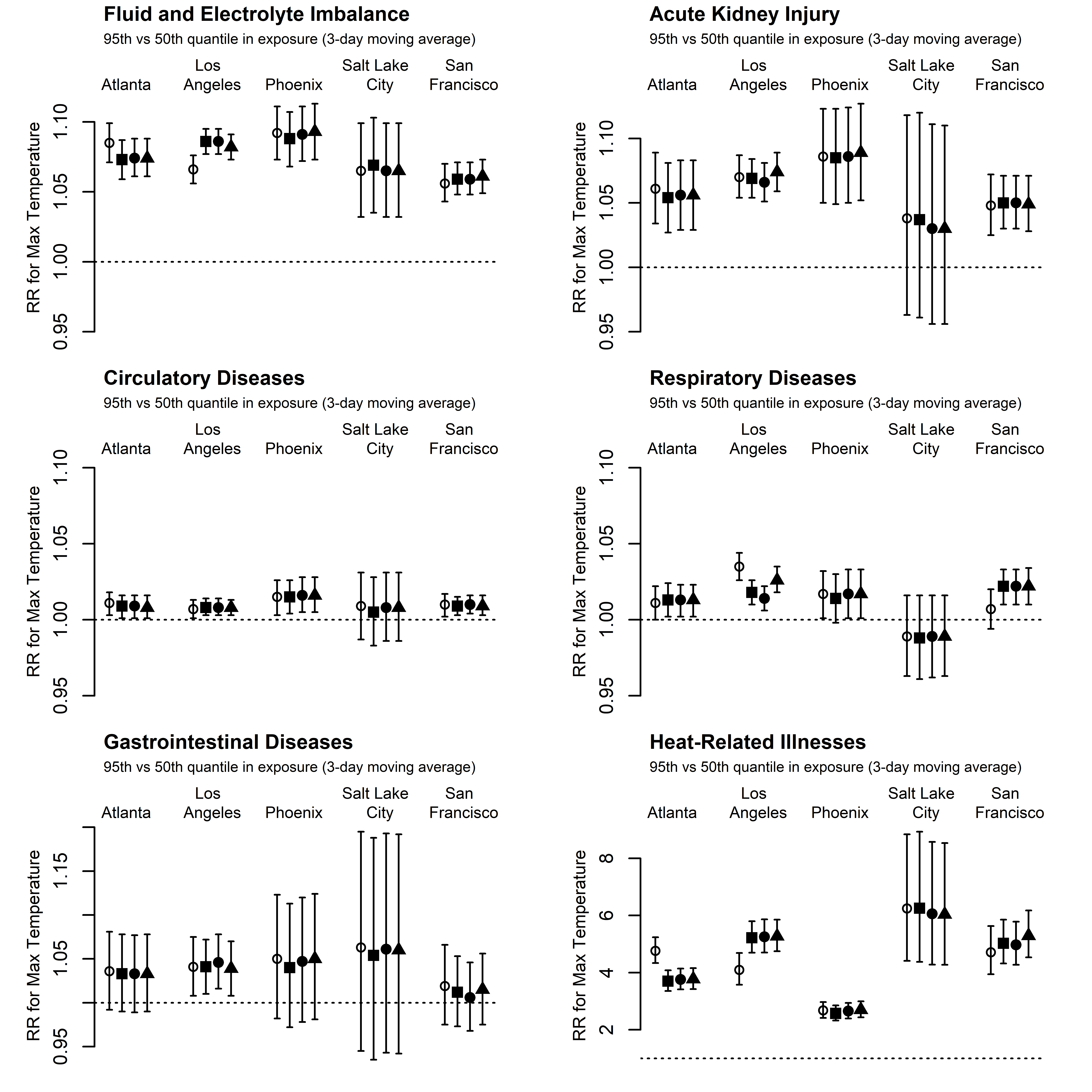
**
